# Brown tumor in a patient with hyperparathyroidism secondary to chronic renal failure

**DOI:** 10.1590/S1808-86942010000300022

**Published:** 2015-10-20

**Authors:** Marlene Correa Pinto, Scheila Maria Gambeta Sass, Cláudia Paraguaçu Pupo Sampaio, Danielle Salvatti Campos

**Affiliations:** 1 Physician, otorhinolaryngologist and head and neck surgeon. Head of the otorhinolaryngology unit of the Santa Casa de Misericórdia (Holy House of Mercy) in Curitiba, PR; 2 Medical resident of otorhinolaryngology in the Santa Casa de Misericórdia, Curitiba; 3 Medical resident of otorhinolaryngology in the Santa Casa de Misericórdia, Curitiba; 4 Physician, otorhinolaryngologist. Irmandade da Santa Casa de Misericórdia (Sisterhood of the Holy House of Mercy) Curitiba – PR

**Keywords:** hemodialysis, hyperparathyroidism, mandible, osteitis fibrosa cystica, tumor

## INTRODUCTION

Brown tumors are giant cell focal lesions associated with primary or secondary hyperparathyroidism; it may be invasive in some cases, but it is not potentially malignant.^1^, ^2^, ^3^ Although these tumors are present in primary hyperparathyroidism, cases associated with chronic renal failure (CRF) are being reported with increasing frequency. Brown tumors in CRF patients are an extreme form of osteodystrophy.^2^, ^3^, ^4^ These tumors are more common in long bones, ribs and the pelvis, but may occur in any bone, such as the jaw, albeit rarely in this case.^5^,^6^

## CASE REPORT

**Figure 1:**
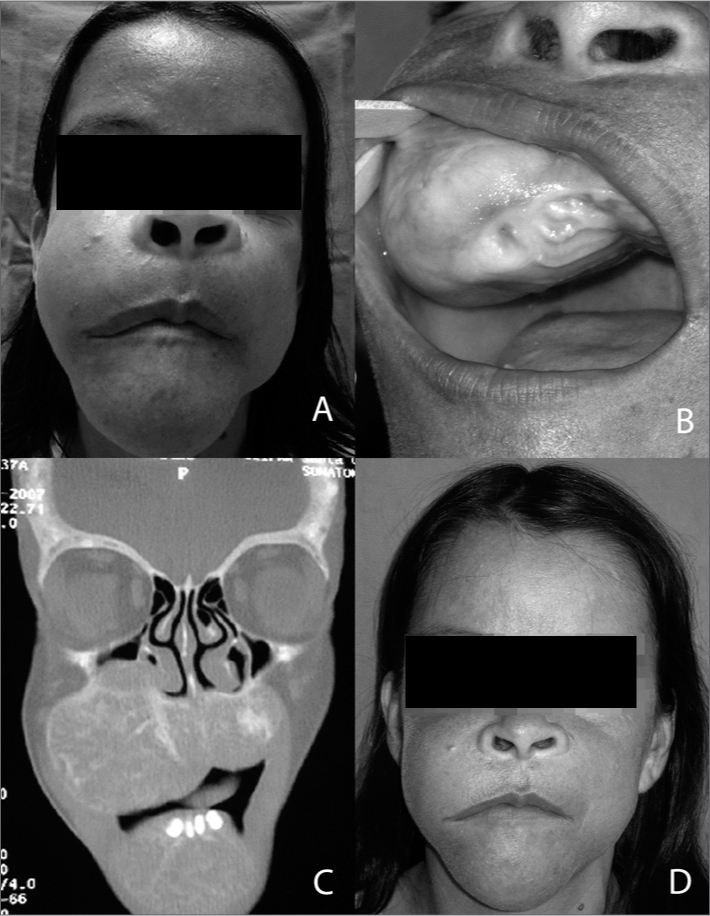
A: Facial asymmetry due to a brown tumor. B: Tumo extending to the palate. C: Coronal computed tomography de monstrating a tumor in the jaw bilaterally, D: Aspect of the fací after parathyroidectomy.

A female patient aged 37 years complained of a painful tumor bilaterally on the jaw and in the mouth. It had started four months before as a small gingival lesion that was treated with curettage by a dentist; however, its volume increased progressively. The patient was on hemodyalisis for the past eight years due to CRF. The physical examination showed a bilaterally enlarged jaw, more to the right, extending significantly into the palate (Figs. 1A and 1B).

Laboratory work up showed elevated creatinine, urea, alkaline phosphatase (1831.0 U/L) and parathormone or PTH (1927.0 pg/ml; normal range 12 to 72pg/ mL). Total and ionic serum calcium levels were decreased. A radiograph of the face showed expanding cystic lesions in the jaw bilaterally. Computed tomography of the face revealed a diffuse and heterogeneous multiloculated lytic bony lesion, which enlarged the mandible and maxilla bilaterally (Fig. 1C). The parathyroid glands were enlarged on the neck ultrasound. Findings confirming hyperparathyroidism confirmed the diagnosis of a brown tumor.

The patient underwent total parathyroidectomy; pathology revealed the presence of an adenoma. On the sixth postoperative day the tumor could be seen to have regressed significantly (Fig. 1D). At 18 months follow-up, the tumor remains stable, but did not regress any further compared to the first few days after surgery. The current PTH level is 70.43 pg/ mL, and alkaline phosphatase is 190 U/L.

## DISCUSSION

Brown tumors are a form of cystic fibrous osteitis, the end stage of bone remodeling in primary or secondary hyperparathyroidism.^2^ Lesions are located in areas of intense bone resorption. Defects become filled by fibroelastic tissue, which deforms the bone and simulates a neoplasm; 1 they should thus be differentiated from other bone tumors of the face, such as true giant cell tumors, giant cell granulomas, and aneurismatic bone cysts.^6^

Parathyroid gland hyperplasia may often be found in CRF patients. Brown tumors are a rare complication of secondary hyperparathyroidism in CRF;4 however, its occurrence has tended to increased because of more prolonged survival rates among CRF patients.^5^

Hypocalcemia, hyperphosphatemia and calcitriol deficiency may be founding CRF, and are the main reasons for increased PTH secretion; this in turn results in morphological changes in parathyroid glands.^4^ PTH alters the intra- and extracellular calcium ratio, thus increasing bone resorption, decreasing bone density, and causing soft tissue calcium deposits. PTH levels in this patient were 26 above the normal reference value; elevated alkaline phosphatase and phosphate, and decreased calcium levels added to a situation of severe osteodystrophy.

Brown tumors occur more often in the mandible compared to the maxilla; they are also three times more frequent in women aged over 50 years. Symptoms may include pain, stiff edema, altered masticatory function, facial deformities (as with the patient in this case), or may be fully asymptomatic.

The therapy of choice is to control the hyperparathyroidism. Tumor regression or complete remission following parathyroidectomy has been well documented In primary and secondary hyperparathyroidism due to CRF. Several authors consider this approach the only correct therapy.^2^ The treatment of choice for the patient in the present case was total parathyroidectomy, after which the tumor regressed within the first few days following surgery.

## FINAL COMMENTS

Brown tumors of the jaw may develop in CRF patients; these tumors may simulate bone neoplasms, and should be included in the differential diagnosis of bone tumors in such patients. Controlling hyperparathyroidism is mandatory, and may be done by carrying out total parathyroidectomy.
